# Comparing Radiologists’ and Artificial Intelligence Performance Detecting Suspicious Microcalcifications on Screening Mammograms: A Pilot Cross-Sectional Study

**DOI:** 10.1177/10732748261470916

**Published:** 2026-07-27

**Authors:** Sarah J. Lewis, Jayden B. Wells, Zhengqiang Jiang, Dania Abu Awwad, Melissa L. Barron, Phuong Dung (Yun) Trieu

**Affiliations:** 1 Faculty of Medicine and Health, The University of Sydney, NSW, Australia; 2 Faculty of Health, Western Sydney University, NSW, Australia

**Keywords:** breast cancer, mammography, microcalcification, observer performance, artificial intelligence

## Abstract

**Introduction:**

Early breast cancer detection through periodic screening is crucial for reducing mortality due to later stage detection. Microcalcifications are common mammographic findings, present in both malignant lesions, benign pathologies, and normal tissues. This study aimed to assess radiologists’ observer performance in determining suspicious calcifications requiring recall, and to compare reader performance to an in-house AI model trained and tested on Australian screening mammograms.

**Methods:**

In this pilot proof-of-concept cross-sectional study, radiologists (n=27), breast physicians (n=2), and final year radiology trainees (n=6), completed the same mammographic test set consisting of 30 mammographic cases displaying different types of calcifications (10 breast cancer, 20 normal/benign). An in-house trained artificial intelligence model (*Sydney-GMIC)* was also applied to the same test set. Performance between readers was compared to AI via Spearman Rank-Order Correlation test. Work experience and caseload trends were compared using independent T tests and Mann-Whitney-U.

**Results:**

Sensitivity was significantly higher in radiologists with ≤ 10 years of experience compared to radiology trainees (72.2% vs 53.3%, *p*=0.042). Furthermore, readers with higher cases read per week (CPW) (i.e. 101-200 CPW compared to 0-20 CPW) had a decreased specificity (58.8% vs 74.6%, *p*=0.041), but higher sensitivity (68.8% vs 59.2%, *p*=0.12). In this pilot dataset, the *Sydney-GMIC* AI model demonstrated higher sensitivity and specificity than the radiologist means. The cases perceived as difficult by AI differed substantially from those challenging for human readers.

**Conclusions:**

This study highlights the challenging nature of recalling calcifications from screening mammograms only, with variable performance among readers. The findings of this small pilot study are exploratory in nature, but the AI performance signals the potential utility of AI models in mammographic analysis of screening cases to progress to recall.

## Introduction

Breast cancer remains the most common cancer among Australian women, accounting for 28% of reported cancers, with approximately 20,500 new cases diagnosed in 2023.^1^ Despite advancements in treatment, breast cancer resulted in an estimated 3,300 Australian deaths in 2023, as reported by the National Mortality Database.^1^ The overall five-year survival rate for breast cancer is 92%, but it can fall to 61% for aggressive subtypes such as for inflammatory carcinoma.^1^ The stage at diagnosis also contributes significantly to survival, with close to 100% survival for Stage 1, but only 32% for Stage 4.^2^ As such, early detection through regular screening is crucial for reducing mortality and improving survival rates, as well as quality of survivorship. Recognising this need, BreastScreen Australia (BSA), a national population-based screening program, provides biennial mammographic screening for women aged 50-74 years.^3^

Microcalcifications are common findings on mammograms, appearing as small opacities. They can be suggestive of premalignant or malignant lesions, however, can also represent benign pathologies or normal variants. The type and composition of microcalcifications are critical for risk stratification; for instance, pleomorphic or fine linear microcalcifications are more strongly associated with malignancy.^4^ One of the most common forms of breast cancer containing calcifications is ductal carcinoma in situ (DCIS), which is often not life-threatening but can become invasive and therefore is clinically important to diagnose.^5^ Most breast calcifications, however, are dystrophic, forming in terminal ductal-lobular units because of processes such as cystic fluid accumulation, infection or inflammation.^4^

Suspicious microcalcifications may be identified based on an assessment of morphology, distribution and stability over time. These include fine linear, fine pleomorphic, amorphous, and coarse heterogeneous types, typically found in segmental or linear distributions.^4^ A greater number of microcalcifications in a small area indicate a high index of suspicion,^6^ whereas microcalcification lesions that have been stable for over two years can generally be considered benign. Hence, it is vital to be able to recognise microcalcification types via mammography, as well as to be able to compare changes over screening rounds.

Standard mammographic views for screening breast cancer, including craniocaudal (CC) and mediolateral oblique (MLO) of left and right breasts, are utilised to visualise the breast tissue comprehensively.^7^ These views can detect masses, architectural distortions, and calcifications.^7^ If women are recalled for assessment based on suspicion, calcifications are often further imaged with magnification views. Magnification mammography provides a closer look at the calcifications, helping radiologists distinguish between benign and suspicious patterns through the morphology and number of calcifications. A short-interval follow-up may be recommended for calcifications that are likely benign, while for suspicious findings, a core needle biopsy with image guidance is often necessary.^8^ While microcalcifications are extremely common (visible on approximately 85% of mammograms), the recall rate reported in an Australian screening cohort was 4.6%, with 0.42% having a biopsy for suspicious microcalcifications.^8^ This study did not report on the cancer type, focussing on the overall radiological interpretation to recall at the screening point.

Emerging evidence suggests that artificial intelligence (AI) approaches in breast imaging have evolved from traditional computer-aided detected systems to more recent deep learning-based classifiers, with convolutional neural networks demonstrating considerable potential for mammography analysis.^9^ Among these, weakly supervised, whole-image classifiers have shown particular promise for screening applications, where lesion-level annotations are limited.^10^ The Globally-aware Multiple Instance Classifier (GMIC), which integrates global and local contextual information, has previously demonstrated strong performance in breast cancer detection on large screening mammography datasets, motivating its evaluation in the specific context of mammographic microcalcifications.^10^ Despite the growing interest in AI for breast imaging, comparatively fewer studies have focused specifically on the detection and interpretation of mammographic microcalcifications, which remain a challenging and clinically important imaging finding.^11^ Additionally, in the development and calibration of AI systems, it is necessary to compare computer performance against that of radiologists, as well as to determine how best to prioritise the trade-off between sensitivity and specificity when using these models. Recent evidence from our group shows that AI systems can exhibit difficulty patterns similar to human readers,^12^ and the present study builds on this body of work by evaluating GMIC in a focused observer-performance framework involving calcification-enriched screening cases and direct comparison with human readers.

The study has three aims. The first aim was to assess readers’ performance to determine suspicious or malignant calcifications requiring recall, while recognising many calcification types can be benign or normal variants and do not require recall. The second aim was to use our in-house AI system, which has been pre-trained for BSA cases, and assess it on the same test-set. The third aim was to compare the AI algorithm with the human readers using various metrics. The images were considered on a case-by-case basis, to elucidate trends in performance among both the human readers and the AI algorithm.

## Materials and Methods

### Study Design and Cases: Human Observer Performance

This was a retrospective pilot observer performance study using reader data from the BreastScreen Reader Assessment Strategy (BREAST) platform between 2023-2024 and received Human Research Ethics Committee approval from the University of Sydney [2019/013]. The study was conducted in accordance with the Helsinki Declaration of 1975.^13^ The readers (n=35 total), who participated in this observational study, included radiologists (n=9 with ≤10 years of experience, n=6 with 11-20 years of experience and n=12 with ≥21 years of experience), breast physicians (n=2) and radiology trainees in their final year who have completed their mammography education (n=6). Each participant completed the same mammographic test-set on the BREAST platform and completed a consent form prior to starting the test-set. The reporting of this study conforms to the STROBE guidelines.^14^

The test-set contained 30 mammogram cases consisting of 10 breast cancer cases and 20 normal/benign cases. Participants were blinded to the proportion of malignant and benign cases but were informed that the test-set is more cancer-enriched than the general population. This test-set was not used during the AI training phase.

The test-set was curated by senior BreastScreen radiologists in NSW who had more than 25 years in interpreting screening mammograms, and they purposively selected cases with both malignant microcalcifications as well as benign calcification. All cases were deidentified. Circles were drawn over the lesions, encompassing all the calcifications, and reviewed by the senior radiologists. The cancer-free mammogram cases were confirmed by at least two senior radiologists with consensus reads in two rounds of normal screening while the cancer cases were confirmed by breast tissue biopsy. Other than the presence of the calcifications, there were no other abnormal features or masses in the curated test set. All cases were screening mammograms with no clinical history provided to participants, and 14 of the 30 cases had prior images, reflective of the variability seen in population-based screening where previous rounds are not always available for every patient. Each case in the test-set contained two mammographic views, a CC and MLO projection for each side. The test-set did not include mammography cases with post-biopsy markers, surgical clips, implants or scars. All cases were obtained from the Cancer Institute New South Wales, who waived the need for obtaining informed consent from the patients whose anonymised mammograms were used in the test-set.

The distribution of breast density in the cancer cases was 30% with A-almost entirely fatty, 30% with B-scattered fibroglandular density and 40% in C-heterogeneously dense whilst 20% of normal cases were B-scattered fibroglandular density, 45% in C-heterogeneously dense and 35% classified as D-extremely dense. Approximately 60% of cancer lesions had a size of equal or less than 15mm.

The participants were asked to read the mammograms in full-resolution on diagnostic monitors ≥5 megapixels and were required to localise all suspected breast lesions for each mammographic projection and rate each lesion in accordance with the Royal Australian and New Zealand College of Radiologists (RANZCR) Imaging Classification: 1 – no significant abnormality (no marking on the mammogram); 2 – benign; 3 – indeterminate/equivocal; 4 – suspicious, or 5 – malignant.

### Data Analysis

The performances of participants were calculated in terms of case sensitivity, specificity, location sensitivity, Receiver Operating Characteristics (ROC) Area Under Curve (AUC) and Jackknife Alternative Free Response Receiver Operating Characteristics (JAFROC) Figure of Merit (FOM). Case sensitivity is a true positive rate measuring the proportion of cancer cases that were correctly marked with a positive rating (3, 4 or 5). Specificity measures the cancer-free cases that were correctly identified cancer-free (rating 1 or 2). Location sensitivity refers to the proportion of each cancer lesion that was correctly localised and identified with positive ratings. It assesses the ability of readers to accurately localise each breast lesion and was determined by correct localisation within the radius of the true cancer lesion which was recorded based on the radiology and pathological reports. ROC AUC evaluates the readers ability to detect cancer cases and identify normal cases, while JAFROC considers the performances of the readers in cancer localisation in association with normal reporting and their ratings. All cancer cases had only one actionable cancer.

### Statistical Analysis

The participants were compared in terms of the above five performance metrics by first comparing participants with varying levels of experience, numbers of cases analysed per week, and whether or not they read for the BSA program. Both independent T-tests and Mann-Whitney-U statistical tests were used for comparison, as appropriate. All statistical analyses were conducted with SPSS and a P value <0.05 was considered significant.

The performance of the participants was then analysed on a case-by-case basis, whereby the percentage of correct and incorrect readers was determined for each case. This was done to determine which cases proved the most difficult by human readers and for later comparison with the AI model (see below).

### AI Model and Analysis

The readers’ performances were compared to cancer case-level probability scores generated by the GMIC model,^10^ which produced saliency maps and incorporates global and local context into its decision-making process. GMIC is one of the highest-performing AI models available in the literature and is publicly available to use and fine tune.^15^ In our previous study, we reported on our technique of transfer learning undertaken using the Australian database (https://www.lifepool.org/) with over 3000 mammograms from Fuji, Sectra, GE Healthcare, and Philips Healthcare vendors. This AI, which our group has called the *Sydney-GMIC,* has demonstrated over 90% in sensitivity and specificity when tested with over 900 curated cases with known difficulty.^16^

Initially, the GMIC classifier used a ResNet-22 network to extract global feature maps, mimicking the global impression radiologists had when reviewing an entire image.^17^ Each global feature map was convolved with a 1x1 filter and subjected to a sigmoid operation, followed by top t% pooling to generate benign and malignant feature maps. Image patches were then selected from these maps based on the largest mean intensity of the patches. Similarly, feature maps extracted from image patches mimic how radiologists focused on suspicious lesion areas including calcifications. These feature maps were then fine-tuned using a ResNet-50 network and weighted via a gated attention mechanism.^17^ The final process combined the global feature map and the attention-weighted representation of image patches to predict image classification. The parameter t for top t% pooling was set to 6.

The model training process was iterative, comparing the accuracy of the models in the current epoch with that of the previous epoch. The number of epochs was set to 100, with training concluding when the validation accuracy did not improve for 3 consecutive epochs. Early stopping occurred at epoch 83 in this study. For uniform transfer learning with the GMIC model, all mammographic images were resized to 2944 × 1920 pixels using bilinear interpolation.^18^ To reduce training time by 5.7%, each mammographic image was tightly cropped to include only the breast region, removing background information using a connected-component labelling algorithm and morphological operations. The model outputted malignant probabilities and saliency maps with the same dimensions as the input images. These saliency maps provide a qualitative visualisation of regions contributing to the model’s prediction. The transfer learning model was trained using the Adam optimisation algorithm with a learning rate of 10^-5, employing binary cross-entropy as the loss function.^19,20^ Both the width and height of the global saliency map were set to 256. The number of patches (K) from the global saliency map for the local module is relatively complex; while classification performance improves with an increase in K, it fluctuates when K > 3. Thus, K was set to 3 in this study.

The cancer probability scores were generated by the *Sydney-GMIC* model for each case. In order to meaningfully compare the AI output to human observer performance, a range of arbitrary AI score ‘cut-off’ values (from 0.1-0.5) were applied, whereby cases with AI scores equal to or above the cut-off were classified as positive for cancer, and cases below the cut-off were classified as negative (benign/normal). The cut-off value from the AI scores was selected to achieve the highest ratio of true positive to true negative rates. This optimised cut-off value was then used to compare the AI’s performance with radiologists’ performance in the same cases. Furthermore, the Spearman Rank-Order Correlation (ρ) was calculated between the returned AI probability value for each particular case and the percentage of human readers identifying the case as cancer. This test was chosen, as the data was found to be not normally distributed by the Shapiro-Wilk test (p=0.11 for percentage of human readers identifying the case as cancer; p<0.001 for AI probability value for each particular case).

## Results

Reader data was collected through an online demographic questionnaire embedded in the BREAST platform, including their current employment, time in current position (mean 18 years, SD=10, for radiologists; mean 4 years, SD=1.5, for radiology trainees), years reading mammograms (mean 16 years, SD=8, for radiologists; mean 7 months, SD=5 for radiology trainees), and number of mammographic cases read per week.

### Reader Analysis

Readers of differing levels of experience were compared in terms of specificity, sensitivity, location sensitivity, ROC and JAFROC. Radiologists’ work position and number of years in current role were used as a proxy for experience in Table 1, whereas case load, determined by number of cases per week (CPW) was used as a proxy for experience in Table 2. The Mann–Whitney U test identified sensitivity as the only significant difference between the groups in Table 1, with radiologists having 0–10 years of experience demonstrating significantly higher sensitivity than radiology trainees (72.2% [95% CI: 59.03, 85.41] vs 53.3% [95% CI: 38.99, 67.67], *p*=0.042). Furthermore, the only significant difference between groups in Table 2 was in specificity, between radiologists completing 0-20 cases per week and radiologists completing 101-200 cases per week, with lower CPW demonstrating higher specificity (74.6% [95% CI: 65.05, 84.11] vs 58.8% [95% CI: 44.65, 72.85], *p*=0.041). No significant differences were observed between the groups working for BSA and those not working for BSA (Table 3).

**Table 1.** Radiologists and Trainee’ Observer Performance With Differing Levels of Experience [Note Breast Physicians Have Been Removed due to Small Numbers]

​		Specificity (%)	Sensitivity (%)	Lesion sensitivity (%)	ROC (%)	JAFROC (%)
Radiology Trainee	N	6	6	6	6	6
	Mean	76.7	53.3	53.3	65.8	65.8
	SD	14.4	13.7	13.7	13.3	13.3
Radiologist (0-10 years)	N	9	9	9	9	9
	Mean	62.2	72.2	71.1	71.0	67.8
	SD	17.0	17.2	17.6	11.3	12.7
Radiologist (11-20 years)	N	6	6	6	6	6
	Mean	77.5	53.3	53.3	67.5	67.5
	SD	15.1	23.4	23.4	10.4	10.4
Radiologist (≥21 years)	N	12	12	12	12	12
	Mean	67.5	61.7	60.8	71.0	68.3
	SD	17.5	12.7	13.1	9.6	11.2

**Table 2.** Readers’ Observer Performance With Differing Numbers of Cases per Week (CPW)

​		Specificity (%)	Sensitivity (%)	Lesion sensitivity (%)	ROC (%)	JAFROC (%)
0-20 CPW	N	12	12	12	12	12
	Mean	74.6	59.2	58.3	68.4	67.4
	SD	15.0	13.8	13.4	11.9	12.3
20-100 CPW	N	7	7	7	7	7
	Mean	67.1	60.0	60.0	66.7	63.7
	SD	21.0	25.8	25.8	7.6	9.0
101-200 CPW	N	8	8	8	8	8
	Mean	58.8	68.8	66.3	69.5	65.9
	SD	16.9	16.4	16.9	11.2	12.8
≥201 CPW	N	8	8	8	8	8
	Mean	74.4	58.8	58.8	71.8	71.3
	SD	14.7	16.4	16.4	10.5	9.8

**Table 3.** Readers’ Observer Performance: Non-BreastScreen Australia (BSA) Readers, BSA Readers Completing 20-150 Cases per Week (CPW) and BSA Readers Completing ≥151 CPW

​		Specificity (%)	Sensitivity (%)	Lesion sensitivity (%)	ROC (%)	JAFROC (%)
Non-BSA Reader	N	15	15	15	15	15
	Mean	71.3	62.0	61.3	69.4	67.5
	SD	15.9	15.7	15.5	11.2	12.3
BSA Reader (20-150 CPW)	N	8	8	8	8	8
	Mean	64.4	57.5	56.3	65.6	63.2
	SD	21.9	23.1	23.3	8.6	8.7
BSA Reader (≥151 CPW)	N	12	12	12	12	12
	Mean	70.4	63.3	62.5	71.1	69.6
	SD	16.3	16.7	16.0	10.7	11.1

### AI Analysis

The performance of the *Sydney-GMIC* on the same dataset is summarised below (Table 4). An independent T-test found a significant difference between the scores assigned to the cancer cases, compared to normal cases, with malignant cases having a higher cancer probability score (0.42 [95% CI: 0.24, 0.60] vs 0.16 [95% CI: 0.08, 0.23], *p*=0.010).

**Table 4.** Summary of AI Cancer Probability Scores Assigned to Cancer Cases, Normal Cases and all Cases, Including Mean, Minimum/Maximum Scores and Standard Deviation (SD)

​	Mean	Minimum	Maximum	SD
Cancer cases	0.42	0.03	0.75	0.25
Normal cases	0.16	0.03	0.48	0.16
Overall	0.25	0.03	0.75	0.23

A range of arbitrary ‘cut-off’ values were chosen (from 0.1-0.5), above which, the cancer probability value returned by the AI program was deemed positive. This enabled the calculation of specificity and sensitivity of the AI program at each probability cut-off value (Table 5), which was then compared to the human readers by means of percentiles as well as one sample T test.

**Table 5.** Sensitivity and Specificity of AI at a Range of Cancer Probability Score Cut-off Values (Arbitrary Scores Chosen, Where if the AI Probability Output for Each Individual Case was Above Cut-off Value, Cases Were Considered Positive for Cancer). Percentile Rows Compare AI Sensitivity and Specificity Scores to Human Reader Data

Probability cut-off value*	0.1	0.2	0.3	0.4	0.5
Sensitivity of AI	0.80	0.80	0.70	0.60	0.4
Percentile of Radiologists	0.68	0.68	0.54	0.36	0.11
Specificity of AI	0.55	0.75	0.75	0.90	1.0
Percentile of Radiologists	0.27	0.54	0.54	0.95	1.0

*AI probability value above which case was considered positive.

Given the superior performance of the AI algorithm using an arbitrary cut-off value of 0.2, the sensitivity and specificity of the AI algorithm at this value was compared to that of the human readers. Overall, AI demonstrated significantly higher sensitivity (80% vs 63%, p=0.043) and specificity (75% vs 68%, p<0.001) compared to the human readers. In terms of correlation, the Spearman Rank-Order Correlation test (ρ) found a weak correlation (ρ = 0.37) between AI probability value and the readers’ accuracy in cancer detection (Table 6).

**Table 6.** Human Reader Percent Correct on Each of Cancer and Normal Cases, as Well as Corresponding AI Cancer Probability Scores Assigned to Cases

​	Case	Human reader % correct (n=35)	AI score
Cancer Cases	1	71.4	0.391
	2	62.9	0.087
	3	42.9	0.502
	4	91.4	0.651
	5	71.4	0.755
	6	97.1	0.449
	7	100.0	0.710
	8	34.3	0.418
	9	11.4	0.210
	10	31.4	0.027
Normal Cases	11	42.9	0.029
	12	68.6	0.440
	13	51.4	0.374
	14	94.3	0.119
	15	62.9	0.062
	16	60.0	0.031
	17	77.1	0.385
	18	37.1	0.047
	19	68.6	0.484
	20	65.7	0.398
	21	74.3	0.042
	22	80.0	0.036
	23	71.4	0.060
	24	80.0	0.182
	25	62.9	0.050
	26	88.6	0.046
	27	68.6	0.126
	28	68.6	0.074
	29	94.3	0.119
	30	71.4	0.068

### Case Analysis

The percentage of readers answering correctly for each case was determined (Table 6). It was found that for 4 of the 10 cancer cases, >50% of the readers answered incorrectly. With regards to the normal cases, it was found that >50% of the readers answered incorrectly on 2 of the 20 cases (Table 6).

**Figure 1.**
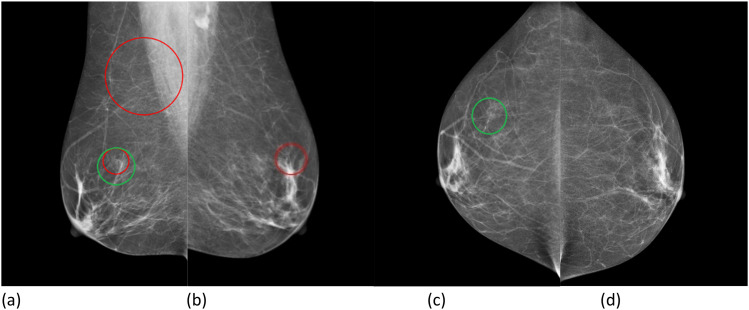
AI performance on Case 2, cancer case, showing lesion location in red circle. Correct cancer location is shown in green circle. (a) RMLO (b) LMLO (c) RCC (d) LCC

**Figure 2.**
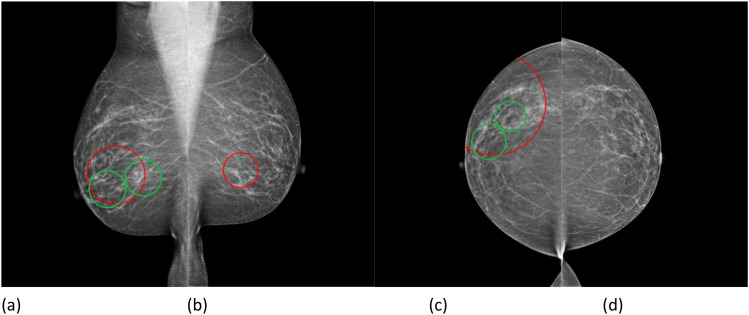
AI performance on Case 6, cancer case, showing lesion location in red circle. Correct cancer location is shown in green circle. (a) RMLO (b) LMLO (c) RCC (d) LCC

The cases that were poorly answered by the AI algorithm (two cancer cases: 2, 10; and 5 normal cases: 12, 13, 17, 19, 20) differed from those that the radiologists performed poorly on (four cancer cases: 3, 8, 9, 10; and two normal cases: 11, 18). Indeed, the only common case that both humans and AI poorly performed was case 10. Figure 1 illustrates the lesion locations and the regions highlighted by the AI model saliency map for case 2 (cancer case). Although 62.9% of readers correctly identified this case, the AI assigned a relatively low probability score of 0.087. Nevertheless, the AI model saliency map highlighted regions overlapping with the annotated lesion on the RMLO view (Figures 1 and 2 shows the lesion locations and corresponding regions highlighted by the AI model saliency map for case 6 (cancer case), which received an AI probability score of 0.449 despite 97.1% of readers correctly identifying the cancer. This case contained two cancer signs, and the relatively modest AI score may be attributed to the overlapping nature of these signs. The model successfully marked both lesion locations.

## Discussion

This pilot study explored the differences in performance among readers when viewing a test-set enriched with calcification cases, using the personal metrics of reader employment, number of cases read per week and BSA reader status. In addition to the human studies conducted, the performance of an AI, the GMIC model with transfer learning for Australian cases, was assessed on the same image set and compared with the human readers, thus giving insight into the utility of AI in mammographic reading.

When comparing readers’ employment/experience and weekly case load, there was few significant differences between radiologists. The decrease in specificity of high-volume readers (101-200 CPW) compared to low volume readers (0-20 CPW) was accompanied by a corresponding increase in sensitivity (59.2 vs 68.8, *p*=0.12), suggesting that those radiologists with a higher case load per week were similarly more cautious in recalling the cases, possibly due to increased clinical exposure. Overall, however, the lack of significant differences between these cohorts in the five metrics being assessed was somewhat surprising, perhaps owing to the complexity of the cases within the image set and the number of true cancers. Indeed, it was found that among 40% of the cancer cases, most of the readers did not identify the case correctly, influencing the lack of significant difference in specificity between cohorts. These results are in partial agreement with Molins et al. which found in a study involving 200 cases and 21 radiologist readers, that among radiologists who conducted routine screening, case volume was not associated with improved performance on screening mammograms, however specificity was found to decrease in radiologists who were not routinely reading mammograms.^21^

Interestingly, no significant differences were found in any of the five metrics in reader cohorts that were split by the number of cases that readers interpreted weekly (i.e. not BreastScreen reader, 20-150 CPW BreastScreen reader, ≥151 CPW BreastScreen reader). This is possibly due to the non-BreastScreen readers having a sufficiently high case load within their own work that they can gain clinical exposure to score similarly to the BreastScreen readers. The lack of differences may also reflect BSA practices of recalling women for further imaging assessment, such as magnification views for calcifications, whereas women who attend private imaging centres may have these views included as part of their visit. This could increase the performance of private radiologists who have more exposure to immediate additional views for calcifications.

With regards to the *Sydney-GMIC* AI model data, there was a significant difference in cancer probability scores between cancer cases and normal cases, however there was marked range in cancer probability scores within each subset. This indicates that while the *Sydney-GMIC* model generally performed well, there was some issues with reliability within both cancer and normal image subsets. In order to meaningfully assess the output of the *Sydney-GMIC* model, a range of arbitrary cut-off values were chosen (0.1-0.5), such that if the AI cancer probability score was above this value, the case was considered positive for cancer. This yielded a series wherein the sensitivity of the *Sydney-GMIC* model decreased with increasing cut-off value while the specificity increased. This was to be expected, as all cases became less likely to be considered positive with an increasing cut-off score. The sensitivity and specificity (80% and 75%, respectively) of the *Sydney-GMIC* model at a cut-off score of 0.2 was found to give the best results. At this cut-off score, the *Sydney-GMIC* model was found to perform within the 68^th^ percentile of radiologists in terms of sensitivity and the 54^th^ percentile in terms of specificity, performing better than the radiologist mean in both metrics. Since these results are within a calcification-enriched dataset, the ideal cut-off score may be different in population-screening settings. Additionally, since the AI probability threshold was selected based on performance within the same dataset used for evaluation, this approach may lead to an overestimation of model performance.

Interestingly, the cases that the *Sydney-GMIC* model performed the worst on varied markedly from those that the humans performed poorly on, except for cancer case 10 (Table 6). In cases 8 and 9 (cancer cases), where human readers performed poorly but the AI had higher performance, it is likely that the faint calcifications were overlooked by less experienced readers who were not attuned to a meticulous search with careful zooming. This potential difference highlights the strength between humans and AI, suggesting that the utilisation of a combined, complementary human-AI screening approach could provide superior screening outcomes. The utility of AI-based detection and classification of microcalcifications has received high attention within literature over recent years, with studies finding various neural networks with the ability to perform at relatively high levels of sensitivity and specificity on microcalcification datasets.^22-24^ For instance, Pesapane et al. described a dataset including 1,986 mammograms, on which the neural network, AlexNet, was able to achieve 0.85 sensitivity and 0.89 specificity for microcalcification classification.^22^ While these studies suggest potential value in having AI as a second reader, further studies could compare AI-aided vs unaided human reading to assess whether AI assistance improves the performance of radiologists.

These results demonstrate the importance of validating AI models against the readers’ performance in detecting abnormal calcifications in a mammogram test-set in order to fine tune cut-off values to suit the purpose being served by the AI model. Comparing the specificity of the *Sydney-GMIC* model at a cut-off score of 0.2, it was found that the sensitivity and specificity was 80% and 75%, compared to the radiologists 63% and 68%, respectively. Given the relatively high performance of AI compared to the mean radiologist specificity and sensitivity in this study, it will likely be important to consider the purpose that the AI model is to serve in specifying cut-off values, based on the trade-off between sensitivity and specificity. For example, in situations where AI is being used as an initial screening tool, higher sensitivity and specificity values are more desirable, meaning that a lower cut-off value is likely to be more useful. Further validation is required as the current results are based on a small, curated pilot dataset and should be interpreted as exploratory.

## Limitations

Several limitations were present within this study, providing avenues for future work and improvement. Within the overall test-set of 30 cases, there were 10 cancer cases, all of which had calcifications. Most of these cases had microcalcifications, some of which were benign as this was the intent of the test-set. As this was a relatively small set, it is possible that the statistical results were somewhat skewed by more challenging cases. Consequently, these results should be viewed as preliminary observations from a curated, calcification-enriched, pilot dataset and may not be directly generalisable to routine population-based screening. Additionally, radiologists within this study lacked the option to peruse or view additional imaging, such as magnification views, which is often undertaken to properly characterise microcalcifications. However, the objective of the study was to measure observer performance from screening images. It would also be beneficial for future work to provide additional images in test-sets so that readers could test their knowledge with additional images before making decisions to progress to biopsy. As access to prior imaging was not available for all cases, this may have influenced reader ability to assess calcification stability. However, this reflects real-world screening practice, where prior examinations are not consistently available for all patients, particularly in cases of first-time screening or irregular attendance.

It can also be argued that some of the cases which were curated as normal for the test-set and therefore not requiring recall, could somewhat be considered indeterminate and readers may have regarded as worthy of recall to biopsy in real life. Calcifications are generally considered more difficult to determine by screening imaging alone and a more experienced reader sample may also assist in clarifying radiologists’ performance, as calcifications are known to be challenging, both perceptually and for clinical decision making. Future studies using larger, more representative datasets will be required to enable adequately powered subgroup analyses of specific suspicious microcalcification morphologies, such as amorphous or fine pleomorphic calcifications, and to further evaluate both human and AI performance across these clinically distinct patterns.

The spread of images from the selected cases were realistic with vendor variations present across the case selection, as well as background parenchymal density. Some of the images, although meeting the technical guidelines of BreastScreen Australia, exhibited lower contrast. The range of contrast can influence conspicuity for the human readers in those cases with moderate background density and the calcifications in the glandular tissue, as opposed to calcifications in the fatty tissue. It is not known how these variations affect the AI probability score as this is very new research.

## Conclusion

This pilot study evaluated differences in observer performance for detecting calcifications requiring recall considered suspicious among varying reader cohorts, considering factors such as position, weekly case load, and BSA reader status. Additionally, the performance of the *Sydney-GMIC* AI model was assessed on the same test-set and compared to the radiologist performance. Within this pilot study, it was found that sensitivity was lower in radiology trainees, compared to those with 0-10 years of experience (53.3 vs 72.2, *p*=0.042), and that higher caseloads (i.e. 101-200 CPW compared to 0-20 CPW) lead to a decreased specificity (58.8 vs 74.6, *p*=0.041), but higher sensitivity (59.2 vs 68.8, *p*=0.12). The *Sydney-GMIC* AI model showed significant differences in cancer probability scores between cancer and normal cases, demonstrating higher sensitivity and specificity than the mean radiologist performance. This highlights the potential utility of AI models in mammographic analysis for microcalcification recall, however further benchmarking research is required, as well as determining optimal AI threshold scores.

## Appendix

### Abbreviations

AI: Artificial Intelligence
AUC: Area Under the Curve
BSA: BreastScreen Australia
CC: Craniocaudal
RCC: Right CC
LCC: Left CC
CI: Confidence Intervals
CPW: Cases Per Week
DCIS: Ductal Carcinoma In Situ
FOM: Figure of Merit
JAFROC: Jackknife Alternative Free-Response Receiver Operating Characteristic
MLO: Mediolateral Oblique
RMLO: Right MLO
LMLO: Left MLO
RANZCR: Royal Australian and New Zealand College of Radiologists
ROC: Receiver Operating Characteristic
SD: Standard Deviation
STROBE: Strengthening the Reporting of Observational Studies in Epidemiology
ρ: Spearman Rank-Order Correlation Coefficient.
